# Brain Waste: The Neglect of Animal Brains

**DOI:** 10.3389/fnana.2020.573934

**Published:** 2020-11-12

**Authors:** Bruno Cozzi, Luca Bonfanti, Elisabetta Canali, Michela Minero

**Affiliations:** ^1^Department of Comparative Biomedicine and Food Science, University of Padova, Padova, Italy; ^2^Department of Veterinary Sciences, University of Torino, Torino, Italy; ^3^Neuroscience Institute Cavalieri Ottolenghi, Orbassano, Italy; ^4^Department of Veterinary Medicine, University of Milan, Milan, Italy

**Keywords:** translational studies, comparative neuroscience, animal cognition, neuro-ethics, farm mammals

The new millennium has seen an explosion of neuroscience research: more than 700,000 articles^1^ have been published on the nervous system, from brain implants to the control of prosthetic limbs to genetic markers to brain plasticity, to name a few expanding fields. It is reasonable to state that we are at the edge of a new era of astounding innovative methodologies and discoveries (see also former US President Obama's NIH speech of April 2, 2013–https://www.youtube.com/watch?reload=9&v=uJuxLDRsSQc). Unsurprisingly, the vast majority of neuroscience investigations have been performed on laboratory rodents (or in cultures derived from their tissues; European Comission, 2013). A relevant number of studies, though, have focused on primates (Grimm, 2018), including apes and man.

The rationale behind the use of laboratory animals (and primates) has been debated countless times, and—to make a long story short—can be summarized by saying that many scientific hypotheses still need to be tested on live mammals, or, at least, on live cells. Tissue cultures cannot replace whole organisms, but, although the limitations are obvious, their use is encouraged for ethical reasons. The choice of the experimental species or tissues to maintain and develop in culture relies on standardized biological parameters, reproducibility of results, management, and other conditions including the availability of the animals and their costs. Although the prevalence of the rodent model in neuroscience has been challenged (Manger et al., 2008; Bolker, 2012; Keifer and Summers, 2016), it still remains the gold standard in translational research for the majority of laboratories. As we are all well aware, the use (some would say sacrifice) of mammalian lives, either directly or to produce cell lines, raises an ethical debate that troubles a large part of the public opinion in the Western world (Bianchi et al., 2018). It is safe to state that, whatever the individual opinion on animal experimentation, nobody is happy about it.

Yet, perhaps, a solution–or at least an improvement of the current situation and the moral weight that the use of lab animals (and specifically mammals) implies–could be nearby and requires a new approach and an innovative mentality. The Western world is moving toward the reduction of environmental pollution, the recycling of materials, and in general toward the reduction of unnecessary waste. Perhaps neuroscience and animal experimentation in Western countries should face that choice too.

We share the world with millions of large-brained domestic farm mammals: cattle, sheep, and pigs are raised for milk and meat in many countries. Millions of horses live in farms worldwide. Domestication of the large herbivores and pigs goes back to the early days of civilization and allowed for the establishment of agricultural societies and progression from the hunter-gatherer lifestyle. Since then, relevant numbers of farm animals are used for meat and milk production, and their organs, including their brains, are available in the millions (see Figure 1).

**Figure 1:**
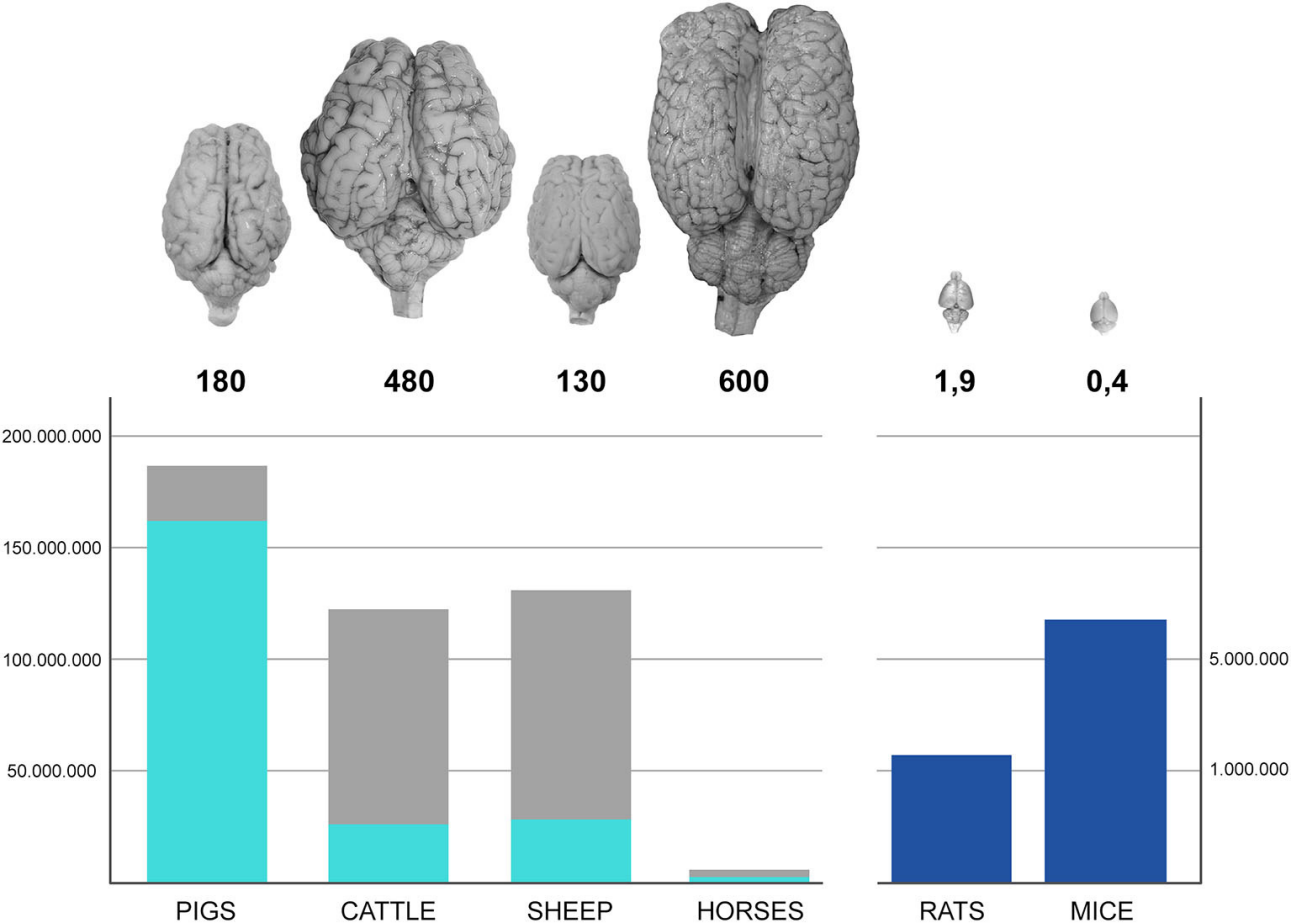
Left bars represent the number (hundreds of millions) of farm animals raised in Europe (2010–2016); the light blue component corresponds to those actually slaughtered for meat production. Dark blue bars on the right represents the number (millions) of rodents used in Europe in research (2010–2016). US data on lab rodents are not readily available since their use is not regulated by a central authority, but directly by local ethical committees. The numbers below the photographs of the brain of each species represent their average weight in grams.

That is why here we pose two questions: How is it possible that–even in the wake of the current explosion of neuroscience research–we surprisingly know so little about the brain, conscious cognitive processes, emotions, and even sensory capabilities of our domestic companion species (Millman, 2013; Higgs et al., 2020; Neave et al., 2020)? And then again: based on the available knowledge, is it possible to use the nervous system of farm animals raised for meat production in neuroscience research (Peruffo and Cozzi, 2014)? Could their nervous tissue replace (at least in part) rodent tissue? The two questions are linked. If we do not know enough on the brain of farm animals, we cannot eventually translate their use to the broader field. Yet, recent discoveries on neuronal resilience and restorative brain functions were based on the use of porcine brains (see the Nature article by Vrselja et al., 2019, and the debate that followed). The question is not only the translatability of data acquired in any experimental species into humans (Sauleau et al., 2009; Mogil, 2019), but also whether the need to investigate other mammals is an ethical issue and a scientific goal.

If we dig into the most common search engines, we find that only very few publications have been dedicated to the brain of the domestic bovine (207), sheep (100), horse (4), or pig (414)^2^. There are indications that the cerebral cortex of Perissodactyls and Cetartiodactyls (including large herbivores, whales, and dolphins) works with a slightly different general organization, because of the prevalence of a less distinct lamination, instead of the well-known six layers typical of rodents and primates (Hof et al., 1999; Cozzi et al., 2017). We also know that the sensory world of farm mammals is partly different from ours: they do not see the same color spectrum, have wide eye fields with only limited stereoscopic capabilities (Ede et al., 2019). Furthermore, horses, cows, and pigs are endowed with an incredibly developed sense of smell, testified by the enormous olfactory bulbs, hippocampus, and related structures. The motor pathways for quadrupedal locomotion require extensive development of the extrapyramidal multi-synaptic tracts (Peruffo et al., 2019). But some of these latter sensory and motor characteristic (vision, sensory perception, reduced stereoscopy, development of the olfaction, prevalence of generators of motor schemes) are also present in rodents.

On the other hand, farm animals have large convoluted brains, the mass, and complexity of which are far closer to the human structure than those of lab rodents, and rival those of the apes. The fetal development of cows and horses is rather long (slightly over 9 months) and the growth and maturation of their brain and spinal cord follows most of the human timetable. Several cellular and molecular mechanisms are well-preserved through phylogeny, and thus domestic mammals may be used as a model for human nervous conditions, such as the sheep for Huntington's disease (Morton, 2018), and the bovine for transmissible spongiform encephalopathies (Asher and Gregori, 2018). Large herbivores cannot substitute laboratory rodents in neuroscience, but an alternative approach to translational medicine that encompasses farm animals may yield new angles and unexpected data. One may speculate that nervous tissue from domestic Cetartiodactyls may represent a potential model to study certain aspects of brain survival under hypoxic or hyperbaric conditions, or the interaction of neural cells with innovative recording devices (Giacomello et al., 2011). To this effect the scientific community may devise sound protocols for sampling nervous tissues from selected specimens within the slaughterhouse under closely monitored conditions even during the normal processing of the carcass. Quality sampling would also provide animal behavior scientists with a direct link to brain functional anatomy.

In addition, remarkable differences do exist in complex biological processes between rodents and large-brained mammals, thus reducing the value of the former to translation [the case of non-newly generated immature neurons present in the neocortex of large-brained mammals but absent in rodents is a recent example (La Rosa and Bonfanti, 2018; Piumatti et al., 2018; La Rosa et al., 2020)]. Finally, we are all aware that research requires animal models that ensure reproducibility, and this means breeds with well-defined characteristics and controlled experimental settings. The genetic background of the most common dairy cow and pig breeds are so standardized to match those of lab rodents, however, the absence of studies in these species has been largely constrained by the feasibility of working and maintaining farm animals in a precise laboratory situation. It is noteworthy that several recent studies (Bailey, 2018) have proven that the standardization provided by laboratory life, far from contributing to the scientific validity of results, might have consequences severely hampering it. Laboratory animals experience significant and repeated stress caused by handling, restraint, and other procedures, as well as the experimental procedures applied to them. Such stress is difficult to mitigate and can result in numerous and pervasive effects on the reliability of experimental data and their extrapolation to humans.

The possibility to grow fully chimeric (human) organs in farm animals is now a debate (Servick, 2019). The concept of “species” in neuroscience may be re-discussed (Knoppers and Greely, 2019) and new ethical issues may come forward, as in the case of deep-brain stimulation (Desmoulin-Canselier and Moutaud, 2019).

One may still argue that the there is more than one intelligence (Bräuer et al., 2020) and the evolutionary distance between primates and hoofed mammals is too large to give significance to laboratory data obtained from tissues sampled from the large herbivores and pigs. Yet the evolution of the mammalian brain is a process that started with the differentiation of the Therapsid in the middle Permian period (roughly 250 million years ago), which brought on the advent of placental mammals (Eutheria, 170 million years ago), and finally led (in a very biased human-centered perspective) to the separation of apes and humans a few million years ago during the Pliocene era. The evolution that gave origin to the order Rodent (that includes most laboratory mammals) took place in the Paleocene era, 50-60 million years ago, roughly during the same era in which odd-toed Perissodactyls and even-toed Artiodactyls became independent clades (late Paleocene 56–66 m.y.a. and early Eocene, 33–56 m.y.a., respectively). Rodents are undoubtedly closer to primates in the evolutionary tree, but at the same time we should consider that bats (Chiropteran) are fairly closer to primates than rodents, but experimental research on bats is—perhaps with the exception of the current investigation into the COVID-19 virus explosion–limited and absolutely not translational. As a synthesis, we could conclude that the evolution of mammals and their nervous system has followed a path largely common to all surviving species. Similarities of the *bauplan* vastly override the specie-specific differences.

Thus, we now face an ethical dilemma, but also consider a potential solution to a wider problem. In more than one sense, we have the moral responsibility to study the brain of farm mammals, to know more about how they perceive the world and feel, and thus disband the disturbing thought that the main reason for not studying them is the fear of discovering that their level of cognitive complexity is too high to raise and then slaughter them. However, there is no option, because studying the brain is key for achieving new insights into behavior, and consequently welfare. There are also important implications from an educational perspective. In 2011, the American Veterinary Medical Association, the Federation of Veterinarians of Europe, and the Canadian Veterinary Medical Association issued a joint statement describing the role veterinarians play in educating others about practices that promote good animal welfare (AVMA Model Animal Welfare Curriculum Planning Group et al., 2017). To ensure veterinarians are better prepared to fulfill this duty, there is a need to include current and consistent information about factors that affect animals' welfare such as the functioning of their brain, cognitive processes, and emotions. And then, on a different level, advances in the study of the neuroanatomy and neurophysiology of large ungulates may be the beginning of the use of their nervous tissues in basic neuroscience research (neuropathology including prion disease, ion channels, cell recording, and administration of compounds in cell culture), an alternative to laboratory animals, and a potential solution to the moral debate that accompanies their use.

## Author Contributions

BC devised the article and planned the research. BC, EC, LB, and MM performed the research. LB realized the image. BC wrote the text and EC, LB, and MM revised it. All authors contributed to the article and approved the submitted version.

## Conflict of Interest

The authors declare that the research was conducted in the absence of any commercial or financial relationships that could be construed as a potential conflict of interest.

## Footnotes

^1^Source: Scopus 2000–2018.

^2^Data 1985–2018 obtained in Web of Science computing “brain” and the name of the species in the title, then limiting to “neurosciences” and “article,” and manually excluding evident confounding factors (e.g., Trojan horse for horse brain).
